# Changes of microbial and metabolome of the equine hindgut during oligofructose-induced laminitis

**DOI:** 10.1186/s12917-020-02686-9

**Published:** 2021-01-06

**Authors:** Maimaiti Tuniyazi, Junying He, Jian Guo, Shuang Li, Naisheng Zhang, Xiaoyu Hu, Yunhe Fu

**Affiliations:** Department of Clinical Veterinary Medicine, College of Veterinary Medicine, Jilin University, Changchun, Jilin Province 130062 People’s Republic of China

**Keywords:** Equine, Laminitis, Gut microbiota, Metabolomics

## Abstract

**Background:**

Laminitis is a common and serve disease which caused by inflammation and pathological changes of the laminar junction. However, the pathologic mechanism remains unclear. In this study we aimed to investigate changes of the gut microbiota and metabolomics in oligofructose-induced laminitis of horses.

**Results:**

Animals submitted to treatment with oligofructose had lower fecal pH but higher lactic acid, histamine, and Lipopolysaccharide (LPS) in serum. Meanwhile, oligofructose altered composition of the hindgut bacterial community, demonstrated by increasing relative abundance of *Lactobacillus* and *Megasphaera*. In addition, the metabolome analysis revealed that treatment with oligofructose decreased 84 metabolites while 53 metabolites increased, such as dihydrothymine, N3,N4-Dimethyl-L-arginine, 10E,12Z-Octadecadienoic acid, and asparagine. Pathway analysis revealed that aldosterone synthesis and secretion, regulation of lipolysis in adipocytes, steroid hormone biosynthesis, pyrimidine metabolism, biosynthesis of unsaturated fatty acids, and galactose metabolism were significantly different between healthy and laminitis horses. Furthermore, correlation analysis between gut microbiota and metabolites indicated that *Lactobacillus* and/or *Megasphaera* were positively associated with the dihydrothymine, N3,N4-Dimethyl-L-arginine, 10E,12Z-Octadecadienoic acid, and asparagine.

**Conclusions:**

These results revealed that disturbance of gut microbiota and changes of metabolites were occurred during the development of equine laminitis, and these results may provide novel insights to detect biomarkers for a better understanding of the potential mechanism and prevention strategies for laminitis in horses.

**Supplementary Information:**

The online version contains supplementary material available at 10.1186/s12917-020-02686-9.

## Background

The domestication of horses occurred 5000–6000 years ago and has become an important aid for humans in battle, transportation and trade throughout the history [1]. Now horses are used for their specific qualities such as speed and jumping ability in competitions all over the world. Thus, diseases in horses, especially sports related ones, such as laminitis, have been becoming increasingly important and bringing mental, financial, and technical challenges to horse owners and veterinary clinicians. Recent years our studies in equine laminitis have been focused mainly on gut microbiota, since researcher Lippold S. et al. [2] found that domesticated horses had reduced microbial diversity compared to wild horses, which may be the cause of laminitis.

Laminitis is defined as the loss of attachment between the inner hoof wall and the distal phalanx, which result in the bone to be driven down into the hoof, destroying the surrounding arteries and veins, and finally crushing the dermis and crown of the sole [3]. It is one of the most serious diseases of the equine foot with a total incidences of about 15% of all lameness in the USA, and with over 27 and 4.7% of cases unable to recover and mortality respectively [4]. Laminitis will occur when the attachment between the distal phalanx and inner hoof wall fails. Evidence showed that a large number of factors are contribute to laminitis, including feeding, injury, obesity, and pregnancy, among them, however, the most common reason is overload of carbohydrate due to exposure to certain pastures or excessive grain-based diets [5, 6]. Unfortunately, the exact pathophysiological mechanisms of equine laminitis remain unclear.

The horse gut microbiota facilities digestion and nutrient absorption for host energy production, short chain fatty acid production, and immune health such as protecting against pathogens and diseases. Thus, in horses keeping the gut microbial community and metabolites at a normal and stable condition is crucial for defending a large array of both intestinal and non-intestinal diseases [7–9]. Previous studies suggested that development of equine laminitis is associated with disturbance of the hindgut microbiota and metabolites [6, 10], and several theories have been proposed to expound the changing relationship of hindgut microbial community and metabolites during severe laminitis cases. These hypotheses indicated that occurrence of the lamellar ischaemia was associated with considerable numbers of hindgut-derived vasoactive agents, such as histamines [11], other vasoactive amines [12], or endotoxins, or contributed to the over-production of host enzymes, especially matrix metalloproteinases [13, 14]. Other studies also demonstrated that the development of acute laminitis in horses was pertinent to lactic acidosis resulting from carbohydrate-overloading [15, 16]. Among these theories, one common shared cause of laminitis aetiology is imbalance of hindgut bacterial community which induced by overloading carbohydrate and then it leads to initiate a sequence of events which ultimately contributes to developing of equine laminitis. Equine laminitis model, traditionally, was established by overloading starch in studies. However, the unacceptable incidences of colic and mortality were frequent in this model [15]. After finding the fructans (β-D-fructose polymer with terminal glucose monomers) that could induce onset of laminitis in horses and the symptoms were identical to that induce by starch, thus Pollitt established a laminitis model using commercial fructose polymers (oligofructose) [17].

## Methods

### Animals and treatment

The present study included 10 healthy horses (3 males, 7 mares, age: 6.7 ± 1.06) were purchased form a horse ranch where managed under identical feeding program for the past two years. We also acknowledged that they had not been exposed to any antibiotics or anthelmintic treatment for at least 3 months prior to the experiment. Horses with any digestive diseases or local hoof lesions were excluded in this project. All animals were managed at university stable where they had free access to water and hey for 3 weeks before experiment. The laminitis model was induced by 10 g kg^− 1^ body weight of oligofructose using a nasogastric tube [17, 18].

The microbial changes may result from a direct or indirect effect of lifestyle, dietary habit, genetic, or other factors that vary between individuals. In order to eliminate adverse effect brought by inter-individual variations, a self-comparative analysis was proposed to reduce selection biases and achieve more reliable results. Thus, in this study, the samples collected before treatment were used as a control group and compared with the following treatment procedures. Fecal samples were collected through rectum post-oligofructose administration (POA) and immediately stored at − 80 °C until microbiota analysis. Fecal pH was detected at 4 h intervals in the 0–24 h POA period. Blood samples were collected from jugular vein with EDTA tubes. The horses were euthanized for pathological assessment of the hooves.

### Euthanasia

Three of the horses, one healthy and two laminates, were euthanized for pathological assessment. We used Xylazine-Ketamine composition (IS Abundant Pharmaceutical CO., LTD, Lanzhou China) in the ratios of 1:5 (0.1 ml/kg) as Pre-euthanasia drug with the rate of 0.5–1 ml/second into the jugular vein. Then, sodium pentobarbital (Feilong Pharmaceutical CO., LTD, Heilongjiang China) 0.1 ml/kg was injected via the jugular vein after the veterinary specialist had confirmed that the animals were fully unconscious and completely unable to feel pain which was tested using needle pocked on the surface of ears.

### Hematoxylin and eosin (H&E) and periodic acid-Schiff (PAS) staining

Lamellar tissues were collected within 1 h after euthanasia, and sectioned with a band saw as soon as possible. A 10 × 10 × 0.5 mm section was cut from each hoof which included the hoof wall and lamella tissue. Each specimen was divided into 55-mm-square blocks that were fixed in 4% formalin for 24 to 72 h before performing by routine methods and imbedding in paraffin wax. Care was taken to ensure that the blocks of lamellar tissue were always obtained from the same location for all horses. Sections were stained using H&E and PAS staining methods and then examined under a light microscope according to a previous study [19]. The H&E sections and PAS sections were used for detection of lamellar lesions and for morphometry, respectively. All digital images were taken using image capture software and a microscope-attached camera (Olympus, Japan).

### Doppler Ultrasonographic measurement

In this study, Doppler ultrasonographic measurement was performed following a previous description on the medial digital artery of both healthy and lame horses after 20 h of induction [20]. In control group, this process was performed without sedating the horses while all legs bearing weight. However, in laminitis group, doppler ultrasonographic measurement was recorded in standing/lying position under sedation (xylazine 2 mg/kg IM) due to the severe pain. After adjusting and repeating 3 times each, the averaged diameter for volumetric flow calculation and the diameter of the artery were obtained.

### LPS concentration detection

Blood samples were collected at 0 and 24 h after oligofructose administration. The samples were centrifuged at 14000 g for 30 min at 4 °C, and the supernatants were transferred into a sterile, depyrogenated glass tube. The concentration of LPS were detected by a chromogenic endpoint assay (Chinese Horseshoe Crab Reagent Manufactory Co.,Ltd., Xiamen, China) with a minimum detection limit of 0.01 EU/mL according to the manufacturer’s instructions.

### Lactic acid and histamine concentrations detection

Blood samples were collected at 0 and 24 h after oligofructose administration. The samples were centrifuged at 3000 g for 30 min at 4 °C, and the serum were collected to detect the concentration of lactic acid and histamine using detection kits according to the manufacturer’s instructions (Suzhou Feiya Biological Technology, Suzhou, China).

### DNA extraction, Illumina MiSeq sequencing, and bioinformatics analyses

The genome DNA from feces was extracted by a CTAB/SDS method. The DNA concentration and purity were detected by 1% agarose gels, and the16S rRNA was amplified by barcoded primers (16S V4:515F-806R) targeting the V4 region. The PCR reactions were conducted with Phusion® High-Fidelity PCR Master Mix (New England Biolabs). PCR products were mixed in equal ratios and then purified with a Qiagen Gel Extraction Kit (Qiagen, Germany). Sequencing libraries were generated using the TruSeq® DNA PCR-Free Sample Preparation Kit (Illumina, USA). The library quality was evaluated by a Qubit@ 2.0 Fluorometer (Thermo Scientific) and an Agilent Bioanalyzer 2100 system. Finally, the library was sequenced on an Illumina HiSeq 2500 platform, and 250 bp paired-end reads were generated. Bacterial community diversity and richness were analyzed by ace, chao 1, the shannon index, the simpson index and the observed species. The distance of bacterial community between control and laminitis was evaluated by nonmetric multidimensional scaling (NMDS) of Bray-Curitis dissimilarity. The bacterial taxa differentially between control and laminitis was evaluated by linear discriminant analysis (LDA) effect size (LEfSe), and the Venn diagrams was conducted to evaluate the numbers of core genera in the cecal contents from the control and the laminitis group horse.

### Metabolic extractions

Intestinal contents were resuspended with prechilled 80% methanol and was incubated 1 h at − 20 °C. Then, the samples were centrifuged 14,000 g for 20 min at 4 °C. The supernatants were transferred to a fresh Eppendorf tube and spun in a vacuum concentrator until dry. The metabolite pellets were detected by LC-MS.

### LC-MS analysis

LC-MS analysis was performed by a Vanquish UHPLC system (Thermo Fisher) coupled with an Orbitrap Q Exactive HF-X mass spectrometer (Thermo Fisher) operating in the data-dependent acquisition (DDA) mode. Samples were injected onto a Hyperil Gold column (100 × 2.1 mm, 19 μm) by a 16-min linear gradient at a flow rate of 0.3 mL/min. The positive polarity mode was eluented by eluent A (0.1% FA in water) and eluent B (methanol), and the negative polarity mode were eluented by eluent A (5 mM ammonium acetate, pH = 9.0) and eluent B (methanol). The solvent gradient as follows: 2% B for 1.5 min, 2–100% B for 12 min, 100% B for 14 min, 100–2% for 14.1 min, 2% B for 16 min. Q Exactive HF-X mass spectrometer was conducted on positive or negative polarity model with spray voltage of 3.2 kV, capillary temperature of 320 °C, sheath gas flow rate of 35 arb, and aux gas flow rate of 10 arb.

The raw data files analyzed by UHPLC-MS/MS were used by the compound discoverer 3.0 (CD 3.0, Thermo Fisher) to perform peak alignment, peak picking, and quantitation for each metabolite. The peaks were matched with the mzCloud (https://www.mzcloud.org/) and ChemSpider (http://www.chemspider.com/) database to get the accurate qualitative and relative quantitative results.

### Statistical analysis

Statistical analysis was conducted using GraphPad Prism 6.01 (GraphPad Software, Inc., San Diego, CA). All data were expressed as the means ± SEM. Differences between date means were determined using one-way ANOVA (Dunnett’s t-test) and the two-tailed t-test. A *P* < 0.05 is considered to be statistically significant.

## Results

### Induction of laminitis

**Fig. 1:**
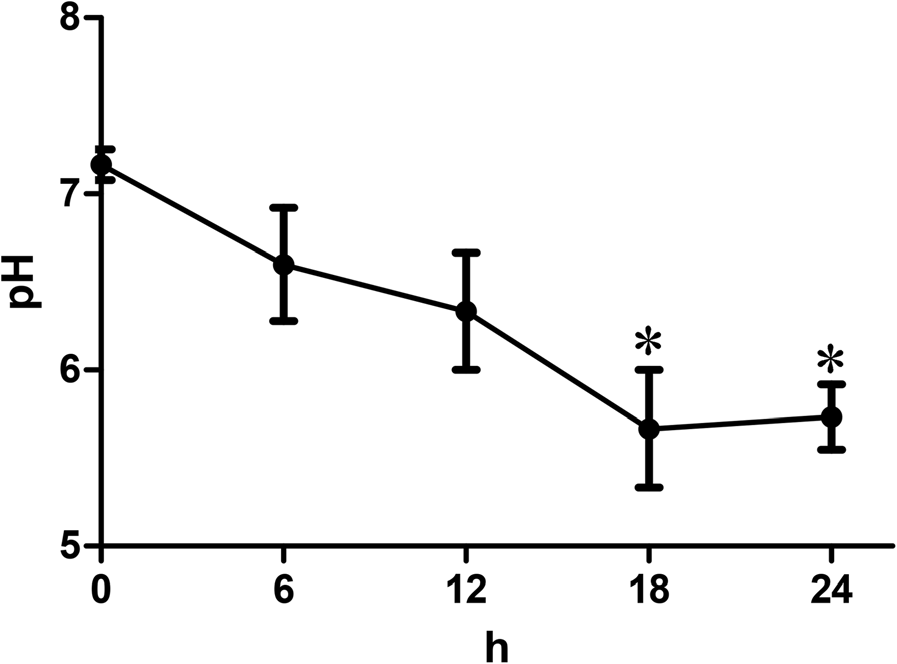
Fecal pH and rectal temperature detection. **a** The fecal were collected and distilled with sterile PBS to detect pH at 6 h intervals in the 0–24 h oligofructose administration period. *P* < 0.05 indicates a significant difference between the two groups

All horses were presented depressed appetite, profuse, and watery diarrhea 8–16 h after administration of oligofructose. Twenty hours later, the horse showed a reaction of weight-shifting and swelling over the coronary band, which were the pathological feature of laminitis. In addition, fecal pH was significantly reduced in the horse that treatment with oligofructose (Fig. 1).

### Effect of oligofructose treatment on lamellar tissue in hooves

**Fig. 2:**
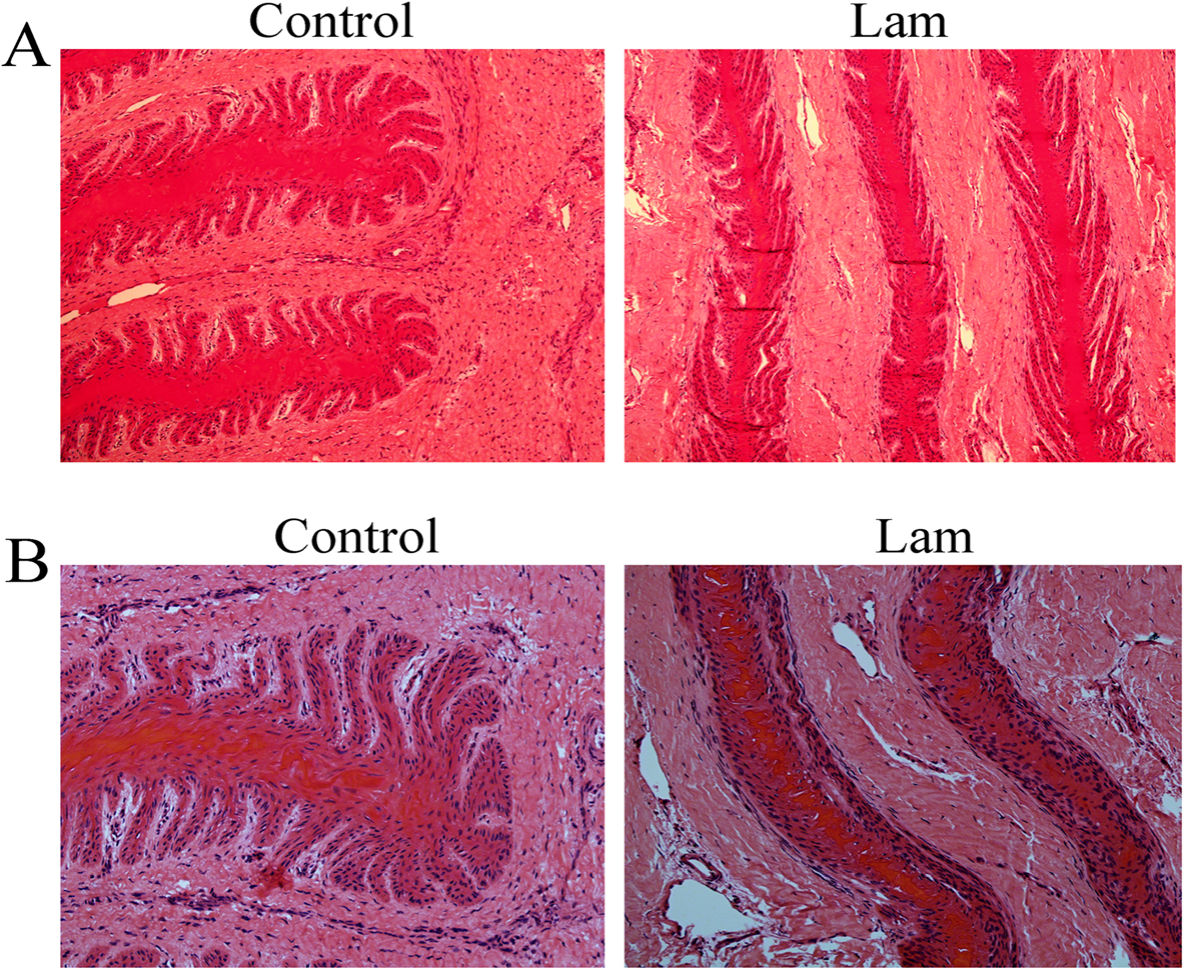
Effect of oligofructose treatment on lamellar tissue in hooves. **a** H&E staining. **b** PAS staining from hoof tissues between control and laminitis horses

As shown in Fig. 2, no lesion was observed in normal hoof tissue sections demonstrated of the lamellar basement membrane. However, discontinuous loss and gaps in H&E and PAS staining was present in the lamellar basement membrane in laminitis horses after 48 h of oligofuctose dosing.

### Doppler ultrasonographic measurement

**Fig. 3:**
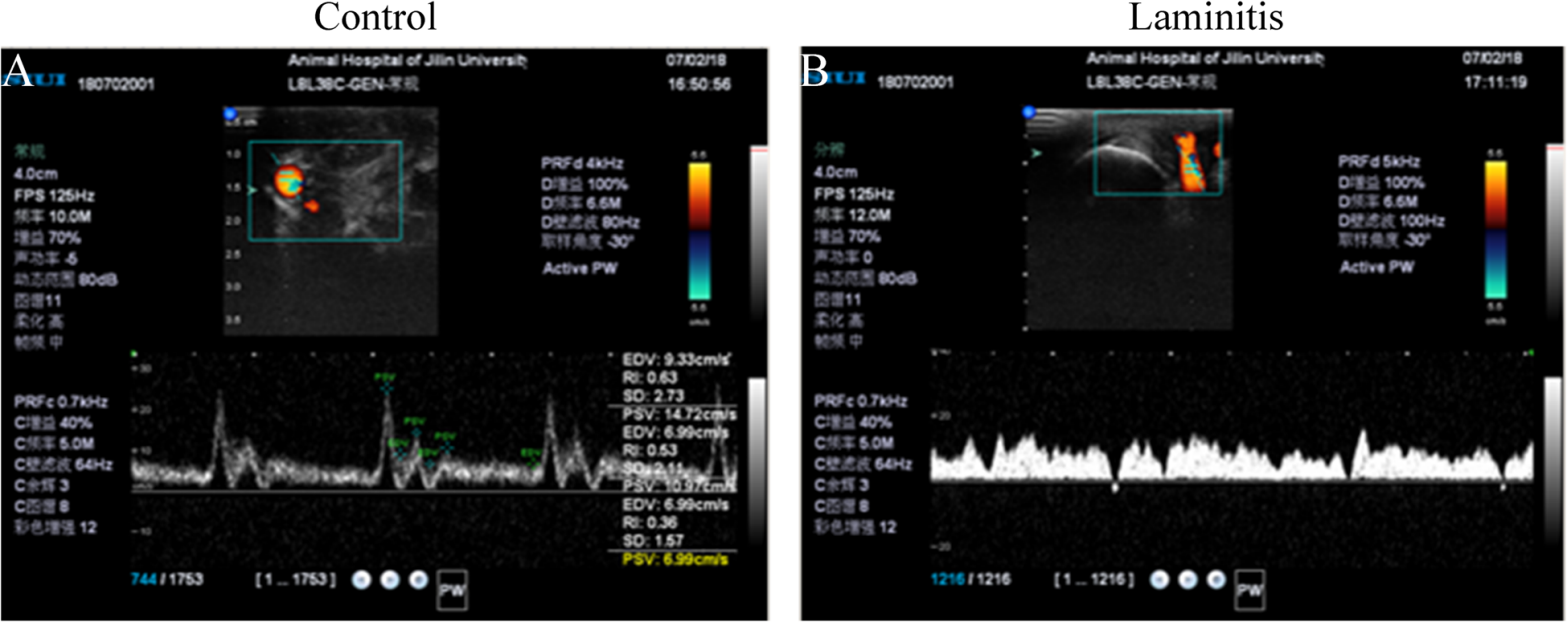
Doppler Utrasonographic measurement. Doppler ultrasonographic measurement was performed on medial digital artery between control and laminitis horses

As shown in Fig. 3a, in control horses, Doppler spectra were showed both high-resistance or low-resistance to nonresistance patterns, which were based on the overall degree of baseline elevation of end diastolic portion of the tracing. However, Doppler spectra of laminitis horses were characterized by low resistance blood flow with low peak systolic velocity (PSV) and low-end diastolic velocity (EDV). The horses suffered laminitis showed a PSV and EDV in doppler spectra decreased, indicating a decrease flow of vascular blood (Fig. 3b).

### Lactic acid, histamine, and LPS in serum

**Fig. 4:**
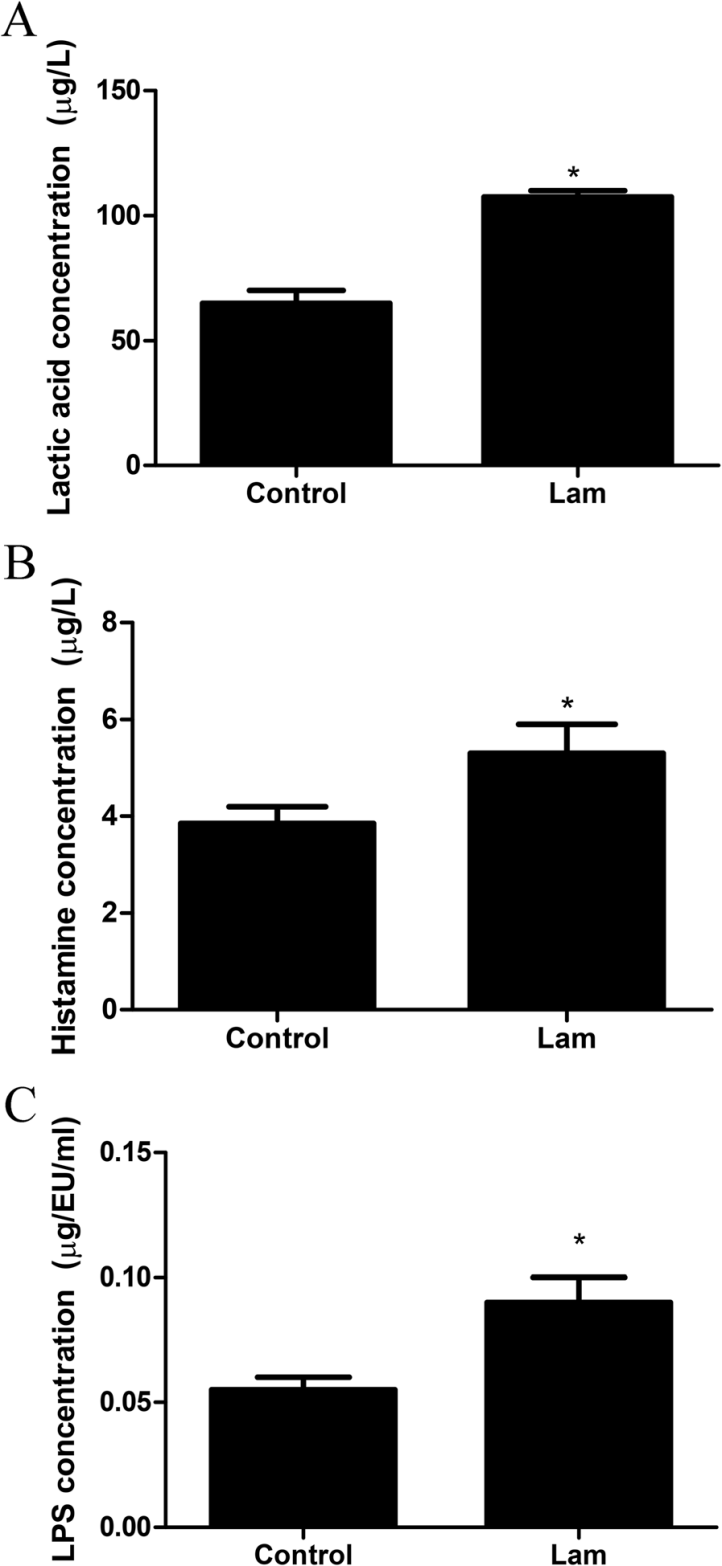
Lactic acid, histamine, and LPS concentrations in serum. **a** Concentration of lactic acid. **b** Concentration of histamine. **c** Concentration of LPS in serum. *P* < 0.05 indicates a significant difference between the two groups

As shown in Fig. 4a-c, treatment with oligofructose obviously increased the concentration of lactic acid, histamine, and LPS in serum.

### Composition of the hindgut bacterial community

**Fig. 5:**
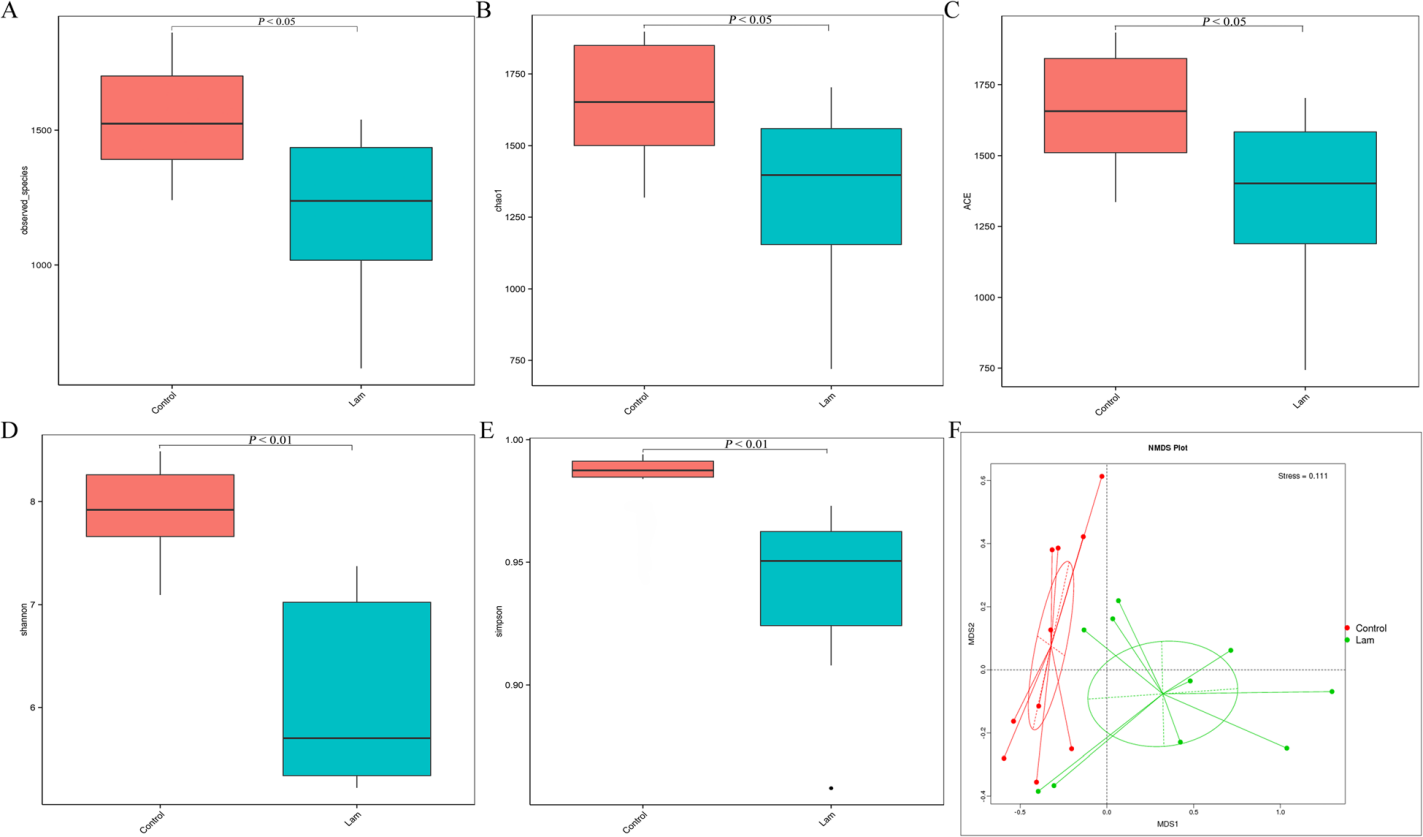
The composition of the intestines bacterial community. Comparison of the microbiota richness in terms of (**a**) observes species, (**b**) chao 1, and (**c**) ace. Comparison of the microbiota diversity in terms of (**d**) shannon index, and (**e**) simpson index. **f** NMDS plot based on Bray-Curtis dissimilarity of fecal microbiota between healthy and laminitis horses. *P* < 0.05 indicates a significant difference between the two groups

Total 1607,927 reads, with an average of 80,396 reads from each sample, were detected using the V4 region of the bacterial 16S ribosomal RNA (rRNA) gene amplified PCR from twenty fecal (10 from control and 10 from laminitis) samples. Rarefaction curves showed that the sampling depth had sufficient sequences to present the majority of bacterial diversity (Fig. S1). The bacterial community richness (observed species, chao 1, and ace) and diversity (shannon index and simpson index) were detected and showed that both community richness and diversity were significantly reduced after treatment with oligofructose (Fig. 5a-e). In addition, NMDS ordination performed on the Bray-Curtis dissimilarity showed that the bacterial community of fecal from healthy horses were separated from those that horses received oligofructose (Fig. 5f).

### Changes of hindgut bacterial community at the phylum level

**Fig. 6:**
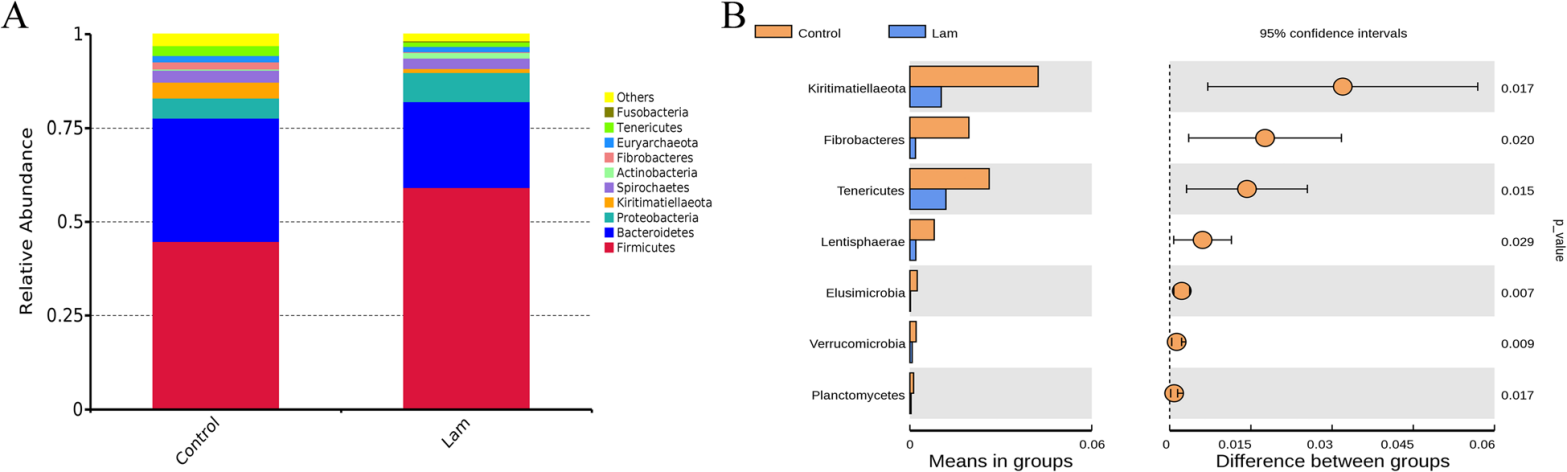
Changes of intestines bacterial community at the phylum level. **a** Relative abundance of the top 10 phyla in the fecal microbiota between healthy and laminitis horses. **b** t-test analysis of different phyla in fecal microbiota at the phylum level. *P* < 0.05 indicates a significant difference between the two groups

At the phyla level, these bacterial sequences obtained from all horses comprised 32 phyla, 28 of which shared in both groups. Among them, *Firmicutes* (control vs laminitis, 44.83% vs 59.17%) and *Bacteroidetes* (control vs laminitis, 32.78% vs 22.84%) were the most abundant phyla in the horse gut bacterial community. In addition, *Proteobacteria* (control vs laminitis, 5.39% vs 7.82%), *Kiritimatiellaeota* (control vs laminitis 4.23% vs 1.03%), *Spirochaetes* (control vs laminitis 3.21% vs 2.77%), *Euryarchaeota* (control vs laminitis 1.67% vs 1.42%), and *Tenericutes* (control vs laminitis 2.62% vs 1.19%) were detected and the relative abundance > 1% in the horse intestines microbiota (Fig. 6a). To assess the changes of fecal microbiota, we compared the relative abundance of the bacterial abundant phyla between healthy and laminitis horses by T-test. The results showed that the relative abundance of *Kiritimatiellaeota*, *Fibrobacteres*, *Tenericutes*, *Lentisphaerae*, *Elusimicrobia*, *Verrucomicrobia*, and *Planctomycetes* were significantly reduced in intestinal microbiota from the laminitis horses when compared with those from the control horses (Fig. 6b).

### Changes of hindgut bacterial community at the genus levels

**Fig. 7:**
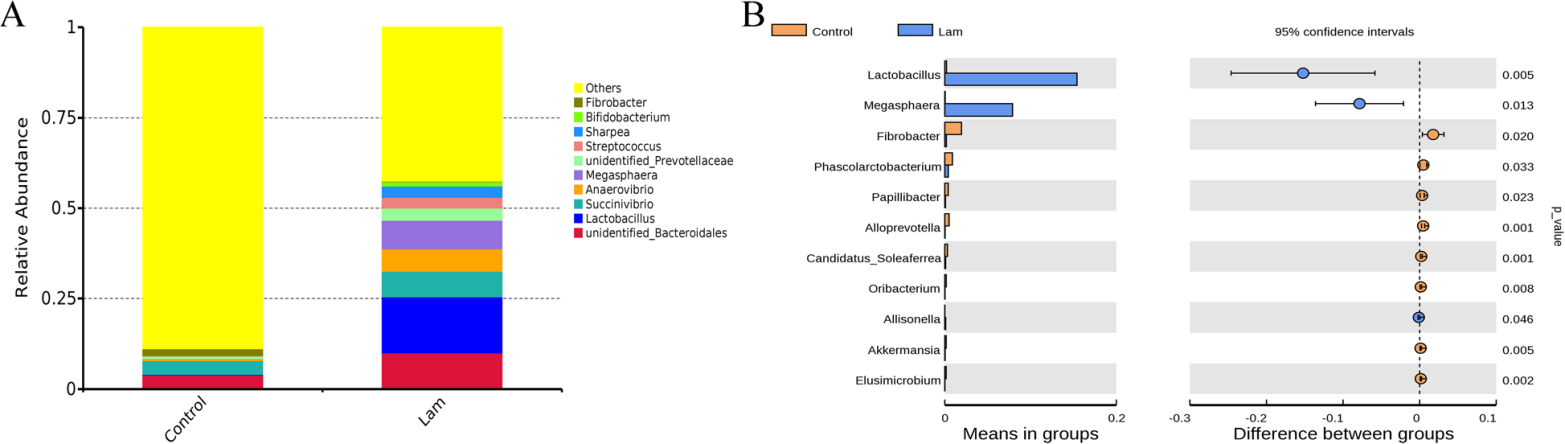
Changes of intestines bacterial community at the genus level. **a** Relative abundance of the top 10 genus in the fecal microbiota between healthy and laminitis horses. **b** T-test analysis of different genus in fecal microbiota at the genus level. *P* < 0.05 indicates a significant difference between the two groups

At the genus level, these bacterial sequences detected from all the animals comprised 265 genera. As shown in Fig. 7a, the dominant genera were *unidentified_Bacteroidales* (control vs laminitis 3.95% vs 10.06%), *Lactobacillus* (control vs laminitis, 0.20% vs 15.42%), *Succinicibrio* (control vs laminitis, 3.76% vs 7.10%), *Anaerovibrio* (control vs laminitis 0.50% vs 6.21%), and *Megasphaere* (control vs laminitis, 0.45% vs 7.90%). Followed by *unidentified_Prevotellaceae* (control vs laminitis, 0.77% vs 3.40%), *Streptococcus* (control vs laminitis 0.43% vs 2.96%), *Sharpea* (control vs laminitis 0.01% vs 3.09%), *Bifidobacterium* (control vs laminitis, 0.1% vs 1.21%), and *unidentified_Ruminococcaceae* (control vs laminitis, 2.78% vs 2.29%). T-test showed that the relative abundance of *Lactobacillus*, *Megasphaera*, and *Allisonella* were significantly increased, while the relative abundance of *Fibrobacter*, *Phascolarctobacterium*, *Papillibacter*, *Alloprevotella*, *Candidatus_Soleaferrea*, *Oribactrium*, *Akkermansia*, and *Elusimicrobium* were reduced in intestines microbiota from laminitis when compared to those from healthy horses (Fig. 7b).

**Fig. 8:**
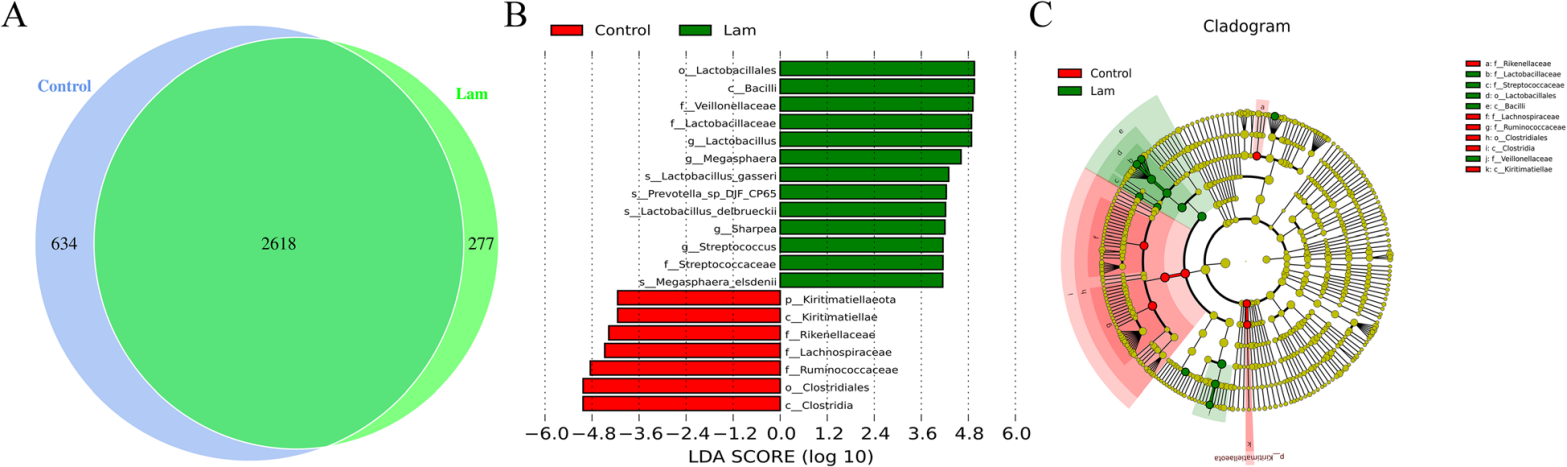
Effect of oligofructose on the composition of the hingut microbial communities in horse at different taxonomic levels. **a** Venn diagram showing the number of core genera in fecal samples from healthy and laminitis horses. **b** Linear discriminant analysis (LDA) score derived from LEfSe analysis, showing the biomarker taxa (LDA score of > 4 and a significance of *p* < 0.05 calculated by the Wilcoxon signed-rank test) in fecal microbiota from healthy and laminitis horses. **c** Cladogram generated from LEfSe analysis showing the relationship between taxon at the levels of phylum, class, order, family, genus, and species. *P* < 0.05 indicates a significant difference between the two groups

Furthermore, the core genera shared by the healthy and laminitis horses were used to evaluate the link between unique bacterial microbiota and laminitis. As shown in Fig. 8a, most genera were shared between healthy and laminitis horse intestines microbiota. As showed core genera accounted for 80.50% of fecal microbiota on healthy horse and 90.43% on laminitis horse. These results suggested that changes in bacterial abundance and interactions among shared bacteria are more important for the development of laminitis. To verify this hypothesis, we performed a biomarker analysis by a LEfSe and a cladogram generated from LEfSe analysis on the microbiota community of intestines. At the genus levels, the biomarkers with significant discriminative power were *Lactobacillus*, *Megasphaera*, *Sharpea*, and *Streptococcs*. At the species levels, the biomarkers with significantly discriminative power were *Lactobacillus_gasseri*, *Prevotella_sp_DJF_CP65*, *Lactobacillus_debrueckii*, and *Megasphaera_elsdenii* (Fig. 8b-c). These results suggested that the bacterial that production of lactic acid play an important role in the development of equine laminitis induced by oligofructose.

### Changes of hindgut metabolites in horse suffering laminitis

**Fig. 9:**
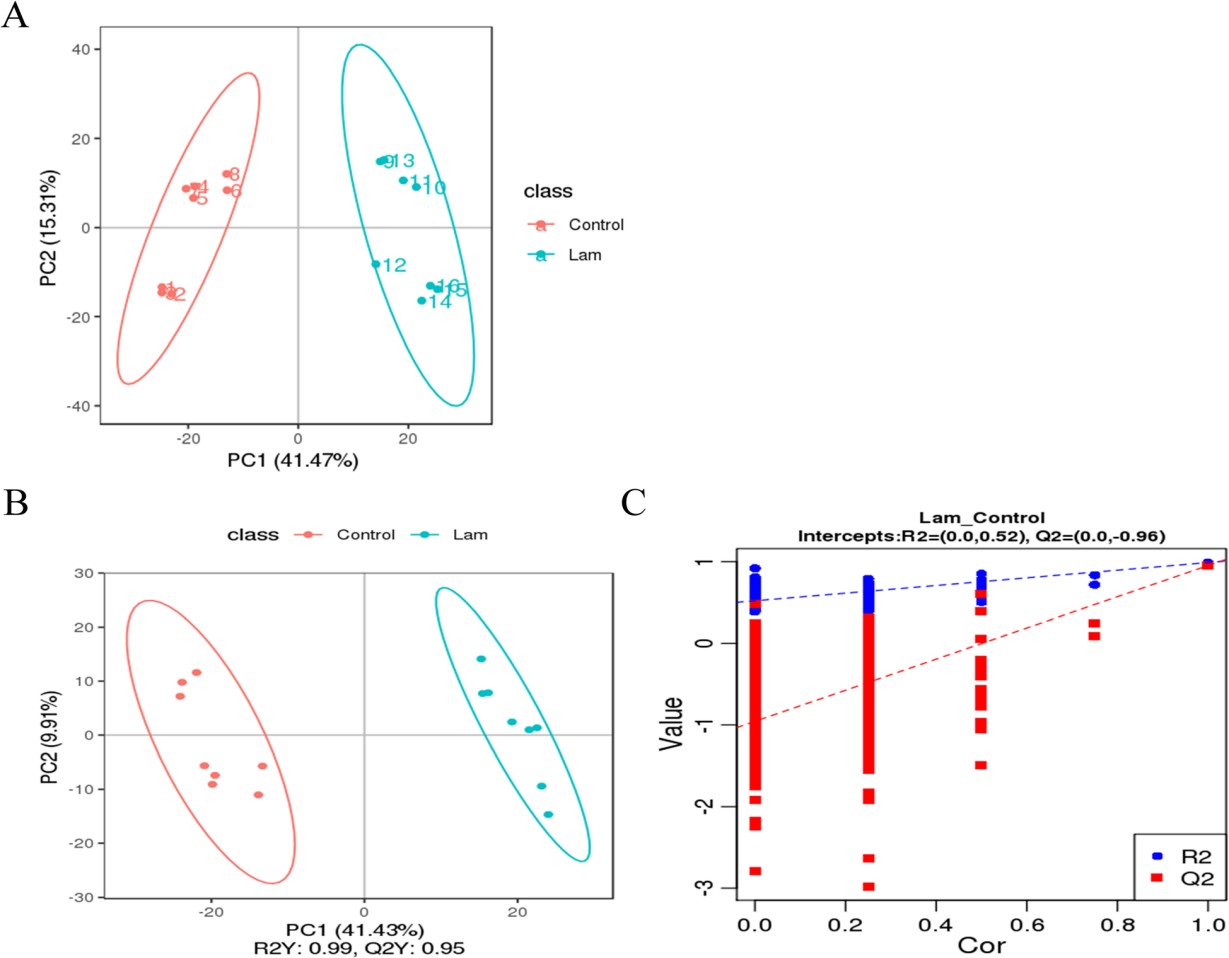
Changes of hindgut metabolites in horse suffering laminitis. **a** Principal component analysis (PCA) of hindgut metabolites of control and laminitis group horses. **b** Partial least squares disrimination analysis (PLS-DA), and (**c**) corresponding validation plots from metabolite profiles of gut samples from control and lamnitis group horses

Principal component analysis (PCA) and Partial least squares-discriminant analysis (PLS-DA) were used to characterize the variations of the metabolic profiles between the control and laminitis group horses in the present study. As shown in Fig. 9a, the distribution of metabolites between control and laminitis horses were obviously separated, and PC 1 and PC 2 accounted for 41.47% and 15.31% of the total variation, respectively. In addition, the OPLS-DA sores results also showed that there were significantly different and distinct metabolite compositions between control and laminitis group horses (Fig. 9b-c).

A total 485 metabolites, mainly fatty acids, lipids, amines, amino acids, organic acids, sugars, nucleosides and other metabolites were identified in the intestinal contents, 137 significantly different metabolites (VIP > 1, FC > 2, and *P* < 0.05), 53 had higher concentrations, such as 10E,12Z-Octadecadienoic acid, asparagine, dihydrothymine, N3,N4-Dimethyl-L-arginine, glycine anhydride, creatine, caprolactam, histamine, N1-(1H-1,2,4-triazol-3-yl)-2-hydroxybenzamide, tiglic acid, 5-Methylcytosine, in laminitis group than the control group. The remaining 84 metabolites were reduced in laminitis group, such as isophorone, D-(+)-Camphor, 4-Ethylbenzaldehyde, 3,5-Dimethoxybenzoic acid, isohomovanillic acid, hydrocortisone 17-valerate, trans, trans-2,4-Heptadienal, phenylpropiolic acid, trans-3-Hexenoic acid, 2-Aminooctanoic acid, and methyl dihydrojasmonate (Table S3).

### Metabolic pathway analyses

**Fig. 10:**
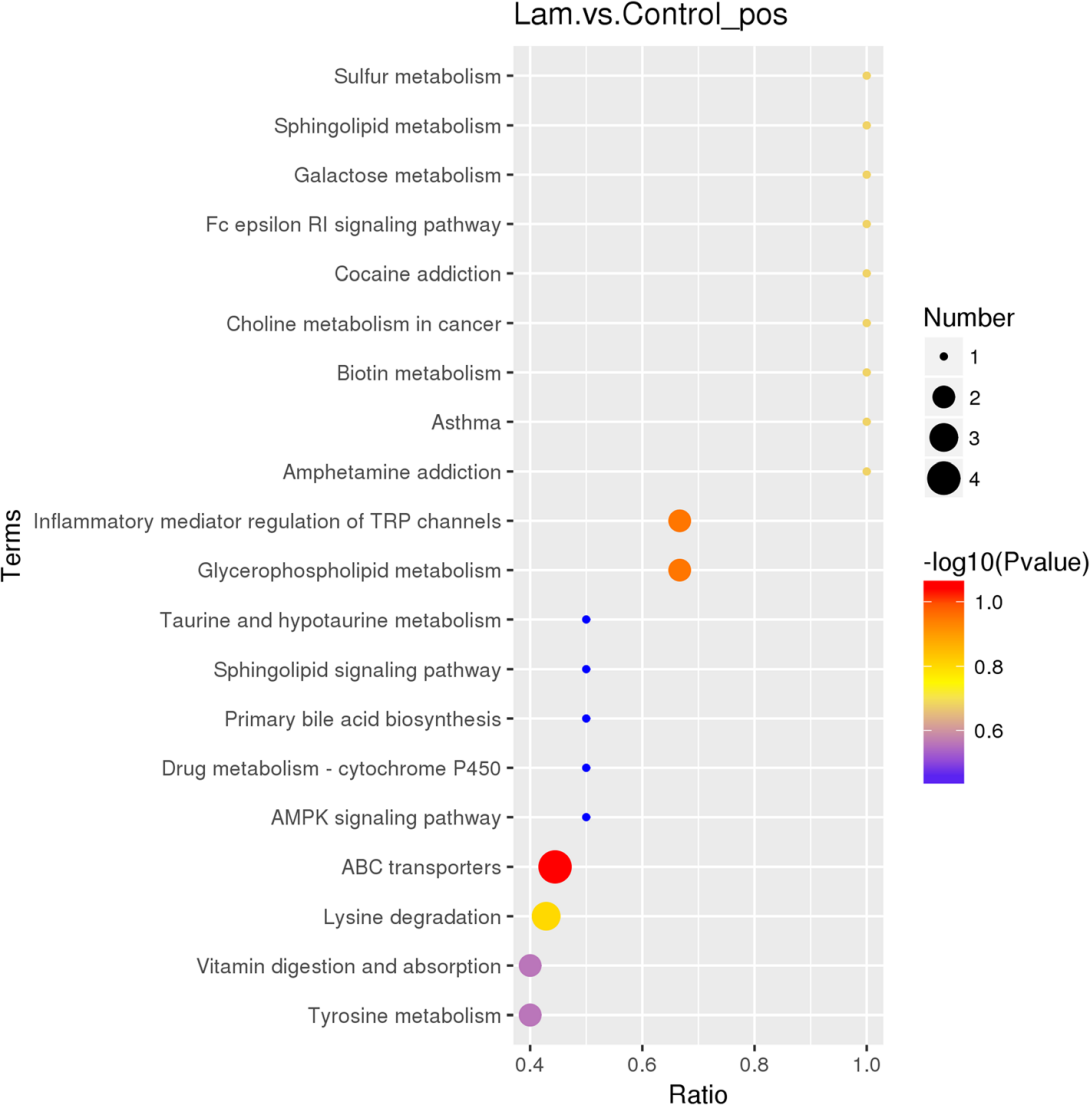
Metabolic pathway analyses. Pathways associated with the metabolites identified in control and lamnitis group horses. The larger size represents higher pathway enrichment, and darker color represents higher pathway impact values

To further understand how multiple pathways changed after treated with oligofructose, analysis of the pathways with differential metabolites were conducted to the Kyoto Encyclopedia of Genes and Genomes (KEGG). As shown in Fig. 10, ABC transporters, glycerophospholipid metabolism, inflammatory mediator regulation of TRP channels, lysine degradation, vitamin digestion and absorption, and tyrosine metabolism were enriched in laminitis group compared with control group horses.

### Correlation between the gut microbiome and metabolome

**Fig. 11:**
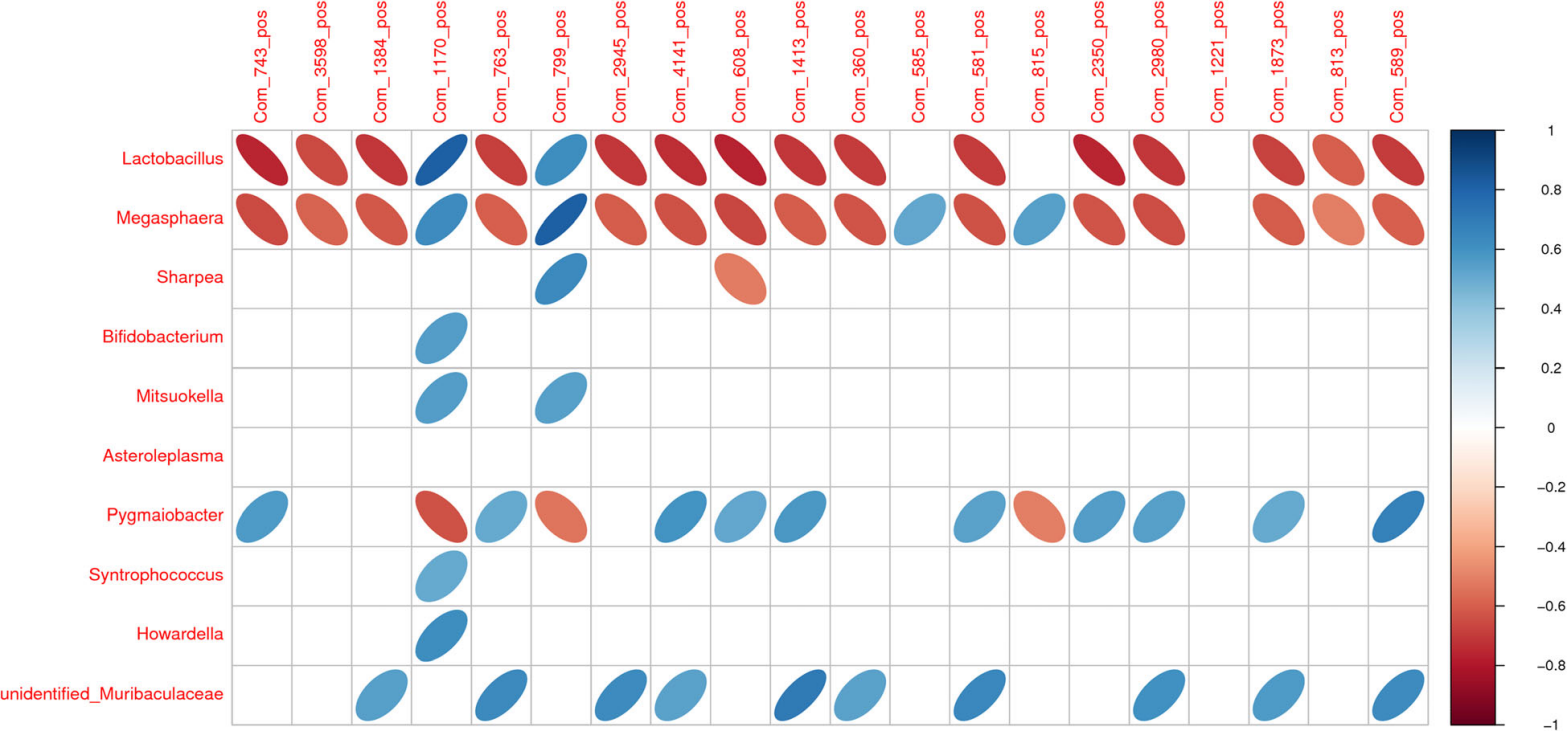
Correlation between the gut microbiota and metabolome. The spearmen correlation network between gut microbiota and metabolome in control and lamnitis group horses

Correlation networks were conducted by means of the data concerning the most abundant taxa (differential genera top 10 between control and laminitis groups) and metabolites (differential metabolites top 20 between control and laminitis groups) created on Pearson’s correlation coefficients (|rho| ≥ 0.6) and *P* ≤ 0.05) in control and laminitis groups (Fig. 11). *Lactobacillus*, *Megasphaera*, *Sharpea*, *Bifidobacterium*, and *Mitsuokella* were the most abundance of the geneus in the gut (Fig. 7a), thus the correlation networks were conducted on In addition to the dihydrothymine and N3, N4-Dimethyl-L-arginine were positivity associated with *Megasphagera*, *Lactobacillus* and *Megasphaera* were both positively associated with 10E,12Z-Octadecadienoic acid, and asparagine, and were negatively associated with isophorone, 3,5-di (2-furylmethylidene)tetrahydro-2H-pyran-4-one, D-(+)-Camphor, 4-Ethylbenzaldehyde, (3beta,9xi)-3-(beta-D-Glucopyranosyloxy)-14-hydroxycard-20(22)-enolide, 4,6,8-trihydroxy-7-methoxy-3-methyl-3,4-dihydro-1H-2-benzopyran-1-one, 2,6-Di-tert-butyl-1,4-benzoquinone, γ-Nonanolactone, 3,5-Dimethoxybenzoic acid, isohomovanillic acid, hydrocortisone 17-valerate, 2,6-Dimethyl-γ-pyrone, trans,trans-2,4-Heptadienal, phenylpropiolic acid, and (S)-(−)-2-Hydroxyisocaproic acid. *Sharpea* was positively associated with asparagine, and negatively associated with 2,6-Di-tert-butyl-1,4-benzoquinone. *Bifidobacterium* was positively associated with 10E,12Z-Octadecadienoic acid. *Mitsuokella* was positively associated with 10E,12Z-Octadecadienoic.

## Discussion

Laminitis is a kind of aseptic inflammation disease in the dermal layers of the hooves, and it causes great pain to the horse [21, 22]. Although the pathological mechanisms of laminitis are still unclear, the role of gut microbiota and its metabolites in equine laminitis is attracting more and more attention [18, 23]. Evidence indicated that the relative abundance of *Lactobacillus* and *Streptococcus* as well as the potentially pathogenic, including *Veillonella* and *serratia,* were increased in laminitis induced by oligofructose or cornstarch [23]. Others also showed that increased the relative abundance of streptococci of the *Streptococcus bovis/equinus* complex may be associated with the series of events which precede the onset of laminitis in the horse [6, 24]. In the present study, we found that the richness and diversity of bacterial community in hindgut were decreased in equine laminitis induced by oligofructose. In addition, the composition of the hindgut microbiota in the healthy and laminitis horses was also significantly different, as showed by the clear separation of sample points from two groups on the NMDS. Generally speaking, the changes were mainly reflected by increasing the relative abundance of genera of *Lactobacillus*, which was the major lactic acid producer. These results were consistent with the research of Milinovich et al., they suggested that *Lactobacillus spp.,* were increased in oligofructose induced laminitis [6]. In addition, the relative abundance of genera *Megasphaere*, a key lactate utilizer [4, 25], also increased, while the relative abundance of *Fibrobacter*, *Phascolarctobacterium*, *Papillibacter*, and *Alloprevotella* were significantly reduced. The others also indicated that increased the relative of *Megasphaere* has been associated with the lactic acid levels recover following starch induction conditions [4]. However, the levels of lactic acid also obviously higher in serum from laminitis horse than the level of lactic acid from healthy horses in the present study. These may be due to the concentrations of lactic acid produced was beyond the utilization ability of the *Megasphaere*. These suggested that the increased numbers of *Lactobacillus* and its metabolite lactate may be one of the most important factors to attribute the development of laminitis.

LPS, the cell wall component of gram-negative bacteria, has been considered to be one of the most important factors in the development of laminitis in horses. It suggested that once carbohydrate overload, a large number of gram-negative bacteria were killed and LPS was released due to the acidic environment which resulted from intaking high carbohydrate feedings. At the same time, the intestinal permeability was increased and then led to a large amount of LPS absorbed into the blood, causing local and systemic inflammatory responses, such as laminitis [21, 26]. Other studies also proved that LPS can cause constriction of peripheral nerve blood vessels, activate the clotting system and cause damage to the hoof of cattle [27]. The present study also showed that the concentration of LPS in serum of laminitis horses were increased when it compared to healthy horses.

Another related theory of the aetiology of laminitis demonstrated that histamine was associated with the development of laminitis in both horses and cattle [28]. Histamine, a vasoactive agent, was shown to accumulated in the horses and cattle gut following by ingestion of high levels of carbohydrates, and led to damage the hoof through circulation [11, 29, 30]. The present study indicated that the concentration of histamine was significantly increased in the serum in laminitis horse. In addition, some bacterial strains, such as *Lactobacillus* [31], have been identified as being able to secrete histamine due to the decarboxylation of histidine through the enzyme histidine decarboxylase activity. The results also showed that the relative abundance of *Lactobacillus*. was obviously increased in the equine intestines bacterial community during laminitis induced by oligofructose. Metabolomics analysis revealed that in addition to the LPS, lactic acid, and histamine, more than 50 metabolitic concentrations were increased in intestinal contents from laminitis horse compared to the control horse. 10E,12Z-Octadecadienoic acid and asparagine not only increased in the horse treated with oligofructose, but also positively associated with the abundance of *Lactobacillus* and *Megasphaera*. Previous studies showed that treatment of 9-hydroxy-10E,12Z-Octadecadienoic acid disrupted epidermal barrier in human keratinocytes [32]. Dihydrothymine is an intermediate in liver pyrimidine catabolism. Studies in animals indicated that changes of pyrimidine metabolism with increased hepatic lipid [33, 34]. Asparagine is a non-essential amino acid in normal cells, as cells are able to synthesize asparagine from other amino acid, such as glutamate/glutamine and aspartate, though transaminases and asparagine synthetase [35]. Studies suggested that depletion of asparagine was associated with improved outcomes in ALI [35]. In addition, obese phenotype was associated with the increase the concentration of asparagine, and others also showed that asparagine is negatively correlated to unhealthy metabolic conditions [36–38]. Arginine is not considered to be an non-essential amino acid for healthy humans, however, it is classified as a semi-essential amino acid with in view of dietary requirement [39]. T-test showed the abundance of *Lactobacillus*, and *Megasphaera* were significantly increased in laminitis group horses when compared to the control group, and correlation analysis showed the *Lactobacillus* and *Megasphaera* both were positively associated with the 10E,12Z-Octadecadienoic acid, and asparagine. Numbers of evidence suggested that the metabolic alterations associated with gut bacterial community disturbance are important biomarkers that indicate the health of human and animals [40–42]. These results suggested that increase of *Lactobacillus* and *Megasphaera*, as well as 10E,12Z-Octadecadienoic acid, asparagine, dihydrothymine, and N3,N4-Dimethyl-L-arginine may be as an auxiliary diagnostic indicator for laminitis in horses.

## Conclusions

In conclusion, we found that oligofructose treatment altered the pattern of hindgut normal bacterial community and metabolites, showed by increased relative abundance of the *Lactobacillus*. *Megasphaera* and levels of lactic acid, histamine, LPS, 10E,12Z-Octadecadienoic acid, asparagine, dihydrothymine, and N3,N4-Dimethyl-L-arginine, which may be associated with the damage of laminar tissues of horses. Hence, targeting intestine microbiota and its metabolites may be an important target for preventing equine laminitis.

## Supplementary Information

**Additional file 1: Table S1**. Relative abundance of all phyla in the fecal microbiota between healthy and laminitis horses. (XLS 28 kb)

**Additional file 2: Table S2**. Relative abundance of all genus in the fecal microbiota between healthy and laminitis. (XLS 28 kb)

**Additional file 3: Table S3**. Differential metabolite screening results (XLS 47 kb)

**Additional file 4: Figure S1.** A rarefaction curve was used to analyze sampling depth to the majority of bacterial diversity.

## Data Availability

The dataset is available from the corresponding author on reasonable request.

## Abbreviations

LPS: Lipopolysaccharide
POA: Post-oligofructose administration
H&E: Hematoxylin and Eosin
PAS: Periodic acid-Schiff
NMDS: nonmetric multidimensional scaling
LEfse: linear discriminant analysis (LDA) effect size
DDA: data-dependent acquisition
PSV: Peak systolic velocity
EDV: End diastolic velocity
PCA: Principal component analysis
PLS-DA: Partial least squares-discriminant analysis
KEGG: Kyoto Encyclopedia of Genes and Genomes
