# Supplementary figures and images for: Changes of microbial and metabolome of the equine hindgut during oligofructose-induced laminitis
Source: BMC Vet Res. 2021 Jan 6;17:11. doi: 10.1186/s12917-020-02686-9 (PMC7789226; doi:10.1186/s12917-020-02686-9)

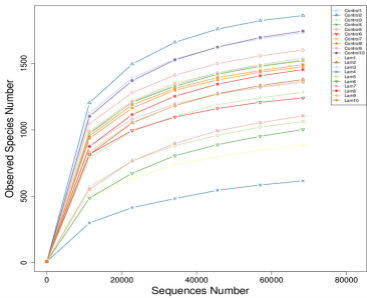

Supplement: Supplementary file 4 — Additional file 4: Figure S1. A rarefaction curve was used to analyze sampling depth to the majority of bacterial diversity. [file 12917_2020_2686_MOESM4_ESM.pdf]
